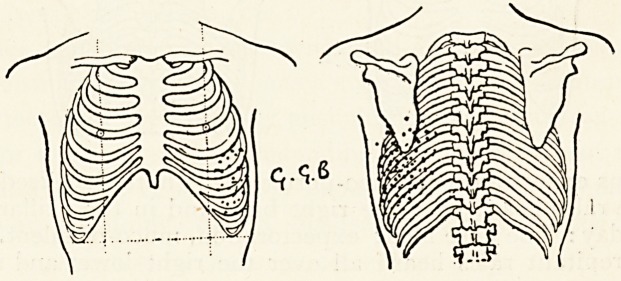# Diplococcal Bronchitis

**Published:** 1902-06

**Authors:** P. Watson Williams

**Affiliations:** Assistant Physician to the Bristol Royal Infirmary, and Physician to Clifton College


### DIPLOCOCCAL BRONCHITIS.

BY

P. WATSON WILLIAMS, M.D. Lond.,

*Assistant Physician to the Bristol Royal Infirmary, and Physician to Clifton College.*

---

WITHOUT attempting to discuss the general question of bronchitis, or to enter deeply into the pathological anatomy of the various forms of bronchitis, I propose to direct attention to a more or less distinct group of cases of acute bronchitis of considerable interest from a medical standpoint.

Up to a very recent period it has been usual to consider acute and sub-acute bronchitis as an inflammatory or vaso-motor phenomenon, induced either by local irritation, as for instance from the inhalation of irritating substances, or from reflex dilatation of the bronchial vessels consequent on a chlll or exposure to cold.

But both exposure to cold and the exposure of the bronchial mucosa to irritating substances by inhalation through the glottis introduce various complicating factors, and one must turn to bronchial asthma or to the paroxysmal bronchitis of infants and young children for the purest examples of vaso-motor bronchitis. Yet it is a remarkable fact that, notwithstanding the recurrent widespread and pronounced bronchial catarrh attendant on paroxysmal asthma, there is, except in old-standing, chronic and complicated cases, but little evidence of those grosser changes which follow acute catarrhal bronchitis. "It is obvious that all diseases, as they fall under the denomination of 'functional,' must proportionately be wanting more or less in those coarser changes in structure that we look for in the study of morbid anatomy; and so it is here. The leading departures are most of them certainly conditioned by, and secondary to asthma of long standing: they are the results of the impaired respiration." (Goodhart[1].)

Contrast with the very pronounced bronchial catarrh of vaso-motor origin,—so acute in its onset and the development of symptoms and physical signs,—the clinical course and morbid anatomy of simple acute bronchitis due to a "chill," arising in young and healthy subjects. It is unnecessary to describe the symptoms, of course; but their gradual development, the general, febrile and gastric disturbances, and the persistence of symptoms for one, two, or three weeks, are strongly suggestive of some infective, inflammatory process, and this is borne out by the widespread pathological changes, with crowding of the smaller lymphatics with leucocytes, enlargement of the bronchial lymphatic glands, etc.

It is not uncommon to meet with such a case as follows:— A child becomes feverish and has a cough; nothing abnormal can be detected in the chest by auscultation or percussion for one, two, or more days, notwithstanding the persistence of the febrile disturbance. Then, often enough not till the general symptoms are subsiding, over a localised area in one lung one

[1] Allbutt's *System of Medicine*, vol. v., 1898, p. 295.

can hear numerous fine râles, sometimes developing evidence of consolidation. Surely it cannot be maintained that this localised bronchitic patch, which may or not pass into lobular pneumonia, and which often does not declare itself till the acuter symptoms and febrile phenomena have subsided, can, *per se*, be the cause of the illness. There is *a priori* reason to believe, rather, that the general disturbance is due to some infective agent, and that the localised lung manifestation is but a consequent lesion of secondary importance. Moreover, the fact that recurrent attacks tend especially to implicate the same area again and again suggests that the infective agent, once it has gained entrance to that area, may lie dormant in the tissues, ready to assert itself when conditions favourable to its renewed activity arise.

Many instances of such localised bronchitis have come under my notice of late, and the frequency with which the sputum is crowded with the diplococcus pneumoniæ or other varieties of diplococci, suggests that these micro-organism are the essential causal factor in a large proportion of such cases. Without entering into wearying details, I would cite the following instances in point :—

CASE.—J. S., boy, aged 16, October 9th, 1901. Symptoms of feverish cold; pain in right side of chest. Temperature rose to 103·2° on the following day. Fine rales, chiefly in right axillary line and the base of the right lower lobe; a few moist sounds heard over whole chest. Pulse, 100; respiration, 24. Fourth day: Temperature fell to 98°, and did not rise again.

Copious expectoration, muco-purulent. A fairly localised patch of fine râles heard over the right base and in the axillary line. Fifth day: The nose bled; expectoration, muco-purulent. The fine crepitant râles heard all over the right lower and middle lobes, but nowhere else. No absolute dulness. Sixth day: Chest rapidly clearing.

Coverslip preparations of the expectoration yielded numerous pure specimens of large encapsulated diplococci, staining Loeffler's methyl-blue and not decolourised by Gram.

CASE.—F., male, aged 58, March 10th, 1901. Was seen by his medical attendant two days before, who made a diagnosis of influenza. The doctor himself fell a victim to influenza, and I saw the patient. His temperature was 100°. The temperature remained above 100° for two more days, and then rapidly fell to normal. He complained of discomfort in the right side of the chest, towards the base, and here a localised patch of fine râles developed and remained for five days. A few fine moist sounds also appeared at the left base.

The expectoration was examined and gave the following results:—*Methyl-blue.* Diplococci and long thin bacilli equally in very large numbers. No streptococci or staphylococci. The diplococci not decolourised by Gram's method. No influenza bacilli on staining with weak carbol-fuchsin and decolourising.

CASE.—G. G. B., boy, aged 12, February 13th, 1901. Had been ailing a few days, and to-day vomited and complained of headache. Temperature normal. At the base of the left lung there is a localised area of fine crepitant râles on deep inspiration, just below the lower angle of the left scapula. The temperature was slightly febrile for a week, not exceeding 99° F., except on two evenings, when it rose to 101° and 100° respectively. The area over which the fine râles were audible increased till it extended over the whole of the lower half of the left lung posteriorly, and extending as far as the anterior axillary line; but over no other part of the chest was there any abnormal physical sign. The area of moist sounds did not clear up till the expiration of three weeks. Muco-purulent expectoration was fairly free for some days and gradually declined.

Expectoration examined:—Coverslip stained with methylene-blue shows an enormous number of two micro-organisms only, the larger, probably saprophytic, being a polar-stained bacillus; the smaller is the diplococcus pneumoniæ, as it stained well with methyl-blue and did not decolourise with Gram's method. Proper staining for the influenza bacillus yielded negative results.

Case.—H. D. S., boy, aged 15½, February 6th, 1901. Has felt poorly one day, with symptoms of slight chill, and this day began to cough. Examination of the chest revealed a localised patch of fine crepitation and sibilant rhonchi immediately below the angle of the left scapula, the rest of the chest yielding no abnormal sounds. Temperature, 100°. From that day onwards the temperature ranged, with occasional exacerbation, between 98° and 99°. The affected area increased in size, but remained localised almost entirely to the left lower lobe, a few fine râles being audible for a day or two at the right base.

Expectoration muco-purulent. After twenty hours' incubation at 37° C. a blood agar slope culture showed numerous dew-like colonies. Staining with carbol-fuchsin showed numerous diplococci. Stained with methyl-blue, numerous diplococci, singly or in pairs, or in short chains; also numerous single cocci. With Gram's method the diplococci were not decolourised. No influenza bacilli could be found. The reporter considered that the diplococci were smaller than diplococcus pneumoniæ.

The patient had a severe and nearly fatal attack of double broncho-pnenmonia in December following.

Case.—W. B., male, aged 38. Attack of bronchitis, with ordinary febrile symptoms, cough and expectoration. Fine and middle-sized râles were heard all over the chest, but were much more numerous at the right base.

Expectoration: coverslip preparations stained for the influenza bacillus gave negative results; but methyl-blue staining showed great masses of diplococci, in places like a pure culture and all of the size of diploccus pneumoniæ. There were no streptococci or staphylococci.

Case.—M. B., boy, aged 18, March 28th. Slightly sore throat, chest clear, some cough. Not till five days had elapsed did he develop physical signs in the chest. His temperature, having varied between 99° F. and 100·6° F., became normal that morning, and it remained practically normal or sub-normal. But on April 2nd numerous fine crepitant rales appeared over the lower half of the left lung, below the scapula. This localised area of moist sounds persisted for six days. There was no area of dulness nor other evidence of consolidation. The expectoration was fairly free for two days before the physical signs appeared in the chest, and persisted for several days.

Bacteriological examination of coverslip preparations were remarkably free from micro-organisms; but revealed diplococci (non-capsulated), staining with methyl-blue and gentian violet, and not decolourised by Gram.

Another group of cases seems worthy of note. Last November four College boys in one small house of 13 boys, four cases of acute bronchitis occurred, two commencing on the 21st, one on the 23rd, and one on the 26th. Among the 19 town boys associated with these there were, at the same time, no less than 13 staying out for bronchial affections, so it was said. Now, my four patients did not have "colds in the head," but from the commencement exhibited symptoms of acute bronchitis, with initial temperatures varying between 102·5° and 103·8°. It might be presumed that climatic conditions were accountable for the occurrence of these cases of bronchitis at the same time; but curiously enough throughout the whole of the rest of the school of 364 boarders there was not a single case of bronchitis during that month, and I have on other occasions observed that cases of acute bronchitis tended to arise in small groups of boys in close association.

The bacteriological results of coverslip preparations of the sputa of these cases were as follows:—

C.—Leptothrix buccalis plentiful; streptococci, numerous long and short chains; diplococci, crowds, non-capsulated.

D.—Diplococci, some capsulated.

Sn.—Numerous capsulated diplococci.

Sy.—Crowds of cocci, singly and in groups (staphylococci) also diplococci in crowds, many capsulated, some in short chains.

Stains in all four methyl-blue, and gentian violet decolourised by Gram. The diplococci in all cases not decolourised by Gram.

Bacteriological examinations of many other cases yielded similar results; but I think the group of cases of which I have given brief notes suffice to support my contention that the diplococcus pneumoniæ is the essential causal factor in many cases of acute bronchitis, and that diplococcal bronchitis tends to affect localised areas in one or more lobes of the lungs, and often runs a very benign course. I submit it is probable that without due care and with exposure to cold such cases may develope ordinary acute lobar pneumonia.

Ritchie [1] examined forty-nine cases of bronchitis and found

[1] *J. Path. and Bacteriol.*, 1901, vii., 1.

pathogenic bacteria in considerable numbers in forty-three (the bacteria in the six negative cases having probably lost their vitality by the time the examination of the sputa was made). He concluded that the most important causal bacteria are the diplococcus pneumoniæ and streptococci, while the influenza bacillus appeared to have been the cause of bronchitis apart from epidemic influenza.

It may be argued that acute lobar pneumonia due to the diplococcus pneumoniæ is a disease with fairly well-defined symptoms running a more or less definite course, with high temperature which eventuates in a crisis generally about the eighth day, and that my bronchitic cases with mild symptoms are so widely different in type and course that it is impossible to regard them as due to the same micro-organism. To this I would reply that enteric fever is in its typical clinical course an equally well-defined infectious disease which runs a fairly well-defined course, but that bacteriological investigations have proved that many of the atypical benign cases formerly known as febricula, and the well-recognised ambulatory type, are true enteric. Moreover, there is good reason to expect that a pneumonic infection of the bronchi and bronchioles will differ materially in clinical manifestations from an infective process of the lung proper. Finally, I would point to the numerous instances of abortive or "larval" pneumonia which, though true diplococcal pnenmomia, are transitional forms between diplococcal bronchitis and ordinary typical acute lobar pneumonia.

Netter[1] himself, who so strongly contends for the pneumo-coccus of Talamon-Frünkel being the specific infecting agent, in 121 *post-mortem* examinations found the primary localisation of the pneumo-coccus in 65.95 per cent. in lobar pneumonia, 15.85 per cent. in broncho-pneumonia and bronchiolitis. The pneumo-coccus has been very frequently found in the catarrhal or lobular pneumonia of children, and it seems as though there is an essential clinical differentiation between an infection of the bronchi and bronchioles and primary infection of the lung parenchyma by this micro-organism.

[1] Cited by Welsh, *Encyc. Med.*, ix. 424.

In acute specific bronchitis the sputum may be crowded with one particular variety of micro-organism, *e.g.* B. influenzæ, streptococci, diplococci, but a few streptococci or a few diplococci are seldom altogether absent. Nor is it safe to assume that preparations exhibiting only a few of such micro-organisms are necessarily derived from the bronchi, inasmuch as both these micro-organisms are so constantly present in the mouths of healthy individuals.

## Figures and Tables

**Figure f1:**
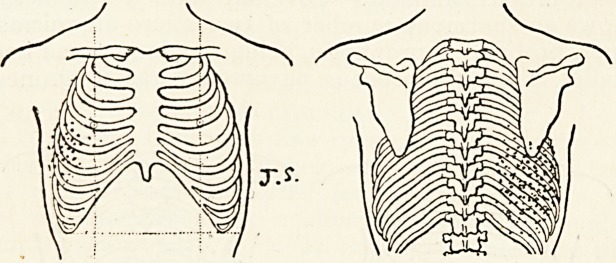


**Figure f2:**